# Morphine may act via DDX49 to inhibit hepatocellular carcinoma cell growth

**DOI:** 10.18632/aging.202946

**Published:** 2021-05-05

**Authors:** Huijun Dai, Jifeng Feng, Zhenhua Nan, Lijuan Wei, Fei Lin, Ren Jin, Suisui Zhang, Xiaoxia Wang, Linghui Pan

**Affiliations:** 1 Department of Anesthesiology, Guangxi Medical University Cancer Hospital, Nanning 530021, Guangxi Zhuang Autonomous Region, China; 2 Perioperative Medical Research Center of Guangxi Medical University Cancer Hospital, Nanning 530021, Guangxi Zhuang Autonomous Region, China; 3 Department of Anesthesiology, Guangxi Maternal and Child Health Hospital, Nanning 530021, Guangxi Zhuang Autonomous Region, China

**Keywords:** morphine, hepatocellular carcinoma, DDX49, anti-cancer

## Abstract

Pain in hepatocellular carcinoma (HCC) is a frequent cause of low quality of life, and morphine is routinely used as a first-line opiate analgesic in HCC. Morphine may exert not only analgesic effects but also anti-cancer effects via unknown mechanisms. Here we show that morphine can inhibit HCC cell proliferation. We further show that DEAD-box helicase 49 (DDX49) is up-regulated in HCC tumors, and that knocking down the DDX49 gene decreases tumor formation *in vivo* and *in vitro,* as well as reduces tumor metastasis *in vivo*. Morphine decreases DDX49 expression in HCC cells. Our results suggest that DDX49 contributes to HCC, and that morphine may exert anti-cancer effects by down-regulating it.

## INTRODUCTION

The prevalence of liver cancer is increasing worldwide. Hepatocellular carcinoma (HCC) is one of the most common causes of cancer-related deaths [1]. Advanced HCC patients often suffer severe pain and other symptoms that reduce quality of life. In fact, 75-90% of HCC patients are in pain at the time of diagnosis [2], and the pain in more than 50% of HCC patients is undertreated. The opioid morphine may be one of the most effective analgesics for HCC patients with severe pain associated with metastasis [3]. Similarly, morphine is a first-line analgesic for managing pain related to many cancers [4]. Opioid analgesics such as morphine are used on the second and third steps of the three-step analgesic ladder proposed by the World Health Organization [5].

In addition to exerting analgesic effects, morphine appears to be able to suppress tumor proliferation in gastric, lung, prostate and breast cancers [4, 6]. Since morphine is metabolized mainly by the liver, it seems reasonable to speculate that morphine may inhibit HCC progression.

Here we provide evidence that morphine does inhibit HCC tumor proliferation and metastasis, and that it exerts these effects in part by down-regulating DEAD-box helicase 49 (DDX49) [7], which we describe here for the first time as up-regulated in HCC. Our results not only identify DDX49 as contributing to the disease, but they describe an anti-cancer pathway through which morphine works, which may guide further research and therapies for HCC.

## MATERIALS AND METHODS

### Patients and tissue specimens

Eighty surgical specimens of HCC tissues and paired adjacent non-cancer tissues were obtained from patients with a mean age of 62 yr (SD, 10 yr) at the Tumor Hospital of Guangxi Medical University between January 2016 and June 2017. Tissue samples were frozen in liquid nitrogen and stored at -80° C. For the present study, HCC and normal liver tissues were confirmed by a pathologist.

Before surgery, patients had provided written informed consent for their clinical samples and medical data to be analyzed and published in an anonymized format for research purposes. The Ethics Committee approved the analysis of the surgical samples.

### Cell culture and reagents

The liver cancer cell lines QGY-7703 and SMMC-7721 were obtained from the Cell Bank at the Shanghai Institutes for Biological Sciences of the Chinese Academy of Sciences (Shanghai, China). The cells were maintained in RPMI-1640 medium (Gibco, Carlsbad, CA, USA) supplemented with 10% fetal bovine serum (Gibco, Carlsbad, CA, USA), penicillin (100 U/ml), and streptomycin (100 μg/ml) at 37° C in a humidified chamber under 5% CO_2_. Morphine hydrochloride was obtained from Humanwell Healthcare Group (Jiangsu, China).

### Cell proliferation assay

We used a commercial MTT assay (Sigma, USA) to assess the proliferation of QGY-7703 cells and SMMC-7721 according to the manufacturer’s instructions. Cells (5×10^3^) were seeded into 96-well culture plates, allowed to attach, then exposed to morphine for 72 h. Cell survival was determined as described [8].

### Wound healing assay

Cells (5x10^5^) were cultured in 6-well sterile plates in complete RPMI-1640 medium. When a confluent monolayer had formed, a sterile 200-μl pipette tip was used to make a scratch across the monolayer in the middle of the plate. At 24 h later, the cells that had moved into the wounded area were photographed under a microscope and analyzed using Image J. Each experiment was performed at least three times.

### Transwell migration and invasion assays

For migration assays, transwell inserts (Corning, NY, USA) were inserted into a 24-well plate. QGY-7703 and SMMC-7721 cells (2×10^4^) were pretreated for 72 h with morphine at 0.01, 10, or 1000 μmol/L, then seeded into the upper chamber. Dulbecco’s modified Eagle medium supplemented with 10% FBS was added to the lower chamber as an attractant for the cancer cells. The culture plates were incubated at 37° C for 24 h. Cells that had migrated through the membrane into the lower chamber were fixed using 4% paraformaldehyde (Santa Cruz, USA), stained with 1% crystal violet (Shanghai Sangon Company, China), and imaged under a microscope (Olympus, Japan). Experiments were conducted three times.

For invasion assays, QGY-7703 and SMMC-7721 cells (2×10^4^) were placed into the upper chamber of transwells in which the membrane had been pre-coated with 50 μl Matrigel (BD, Franklin Lakes, NJ, USA). The plates were incubated for 36 h at 37° C, then processed in the same way as in migration assays.

### Flow cytometry of cell cycle and apoptosis

For cell cycle assays, QGY-7703 and SMMC-7721 cells were pretreated with the indicated concentrations of morphine, then incubated for 30 min at 37° C with 10 μL of RNase A solution (250 μg/mL) and 0.1% Triton X-100. Flow cytometric analysis was conducted using the EPICS XL-MCL FACS system (Becton Dickinson, CA, USA). Data were analyzed using MultiCycle Software for Windows (Phoenix Flow Systems, CA, USA).

For apoptosis assays, QGY-7703 and SMMC-7721 cells were cultured for 24 h, then processed using the Annexin V/FITC Apoptosis Detection Kit (Jingmei Biotech, Shenzhen, China) according to the manufacturer’s instructions. Cells were analyzed using the EPICS XL-MCL system.

### GeneChip analysis

Total RNA was isolated from QGY-7703 cells using TRIzol (Invitrogen, Carlsbad, CA, USA) according to the manufacturer's protocol. Probe synthesis, hybridizations, and analysis of microarray images were performed by Shanghai Genechem (Shanghai, China).

### Lentivirus-mediated overexpression and knockdown of DDX49

Lentiviral plasmids encoding DDX49 (LV-DDX49-OE) or the antisense sequence to target 5'-AGGAGCAGATCAAGAAGAA-3' in the DDX49 gene (LV-DDX49-KD) were prepared by Shanghai Genechem. The antisense sequence knocked down DDX49 expression by at least 90% (data not shown) in QGY-7703 and SMMC-7721.

Efficiency of transfection was measured under a fluorescent microscope based on expression of GFP encoded in the lentiviral plasmids. Transfected cells were seeded into 96-well plate at a density of 2000 cells/well (100 μl per well) and incubated for 24 h at 37° C. The Celigo system (Nexcelom, Lawrence, USA) was then used to assay proliferation in each well every day for 5 days.

### Western blot assays

Total protein was extracted from cell lines using RIPA buffer, and protein concentration was estimated using a commercial BCA kit (Beijing, China). An aliquot (35 μg per well) was fractionated by SDS-PAGE and blotted onto a nitrocellulose membrane. The membrane was blocked with 5% bovine serum albumin (BSA) for 30 min, then incubated overnight at 4° C with anti-DDX49 antibody (1:1000, CST, USA), followed by a secondary antibody (1:1000, CST, USA). Blots were immunostained with antibody against GAPDF (CST, USA) as a loading control. Immunostained blots were analyzed using an imaging system (Alpha Innotech, San Leandro, CA, USA).

### Statistical analysis

Data were reported as mean ± standard error of the mean (S.E.M). Differences between two groups were assessed for significance using Student’s *t* test, while differences among more than two groups were assessed using one-way analysis of variance (ANOVA). Differences associated with p<0.05 were considered statistically significant. All statistical analyses were performed using SPSS 13.0 (IBM, Chicago, IL, USA).

### Ethical approval

This study was carried out in accordance with the Guidelines for Human Specimens for Research in the Guangxi Zhuang Autonomous Region, from the Medical Ethics Committee of Guangxi Zhuang Autonomous Region. The study protocol was approved by the Tumor Hospital of Guangxi Medical University.

## RESULTS

### Morphine suppresses HCC cell proliferation, migration and invasion *in vitro*

**Figure 1:**
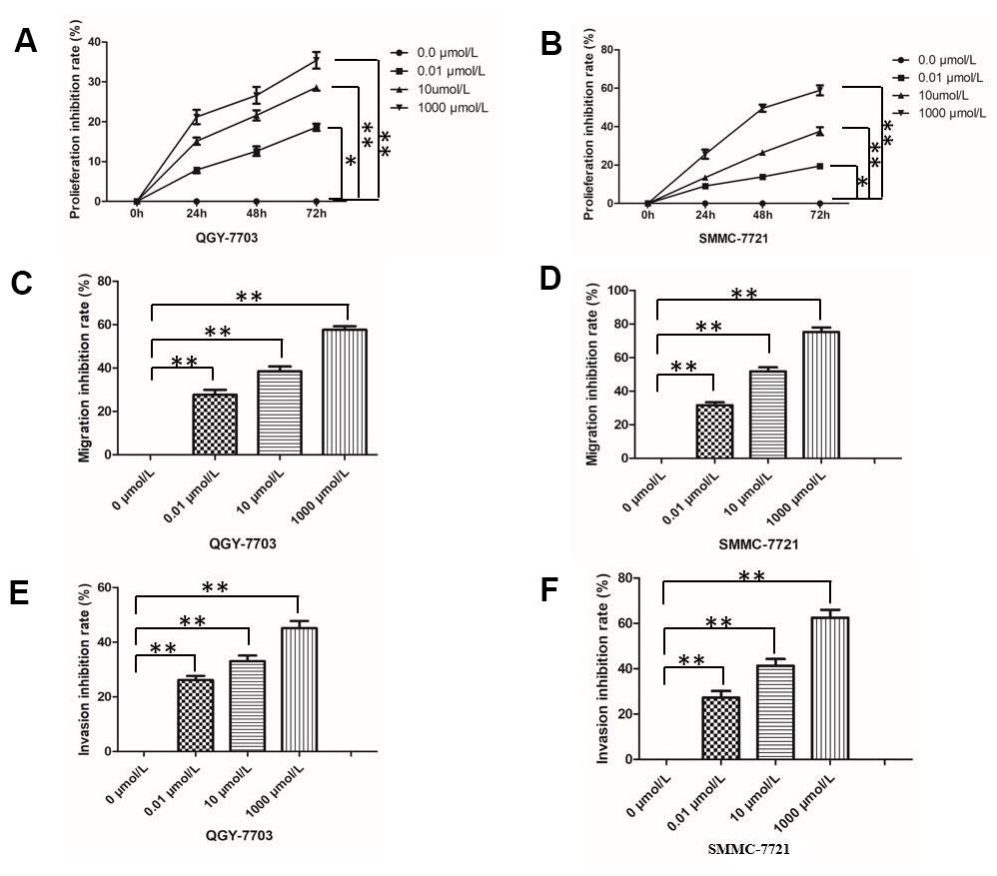
**Effect of morphine on HCC cell proliferation, invasion and migration.** The HCC cell lines QGY-7703 and SMMC-7721 were treated with morphine (0, 0.01,10, or 1000 μm/L) for 72 h. (**A**, **B**) Cell proliferation was assessed by MTT assay. (**C**, **D**) Migration of cells was assessed in a transwell assay. (**E**, **F**) Invasion by cells was assessed in a transwell assay. *P < 0.05, **P < 0.01.

Morphine inhibited growth of the two HCC cell lines QGY-7703 and SMMC-7721 in a dose- and time-dependent manner (Figure 1). At the lowest concentration of 0.01 μmol/L, morphine inhibited growth by 18% (*P* < 0.05), which rose to 40% at the highest concentration of 1000 μmol/L (*P* < 0.05). Morphine also significantly suppressed migration and invasion of both HCC cell lines (Figure 1).

### Morphine arrests HCC cells in G0/G1 and promotes apoptosis *in vitro*

Morphine significantly reduced the percentage of QGY-7703 and SMMC-7721 cells in S phase and increased the percentage of cells in G0/G1, and these effects were dose-dependent (Supplementary Figure 1). At the same time, morphine (10μM) significantly promoted apoptosis.

### Morphine affects the transcriptome of HCC cells *in vitro*

**Figure 2:**
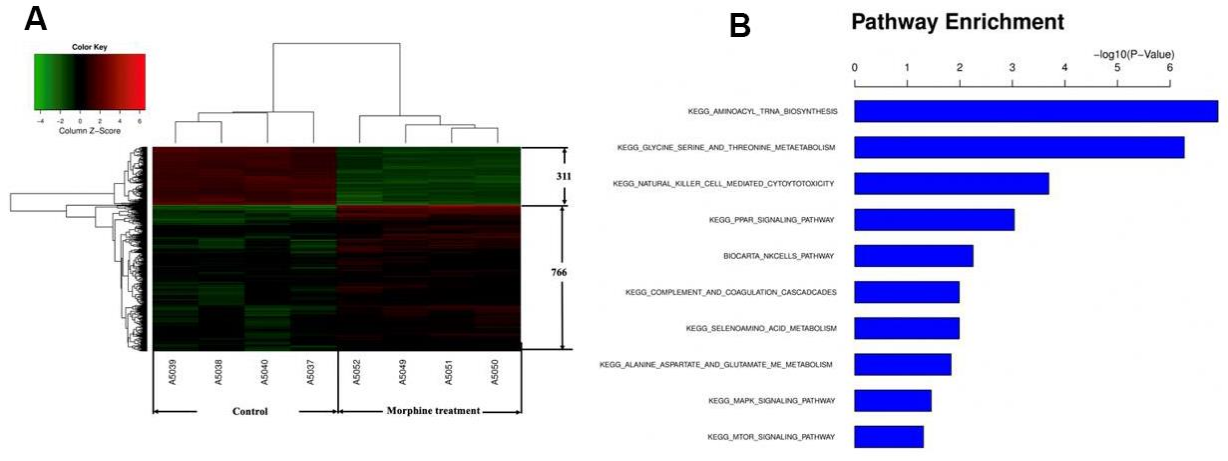
**Genes differentially expressed in HCC cells *in vitro* after morphine treatment.** QGY-7703cells were treated with morphine(10μM) for 48 h. (**A**) Identification of differential gene expression. (**B**) Enrichment of Kyoto Encyclopedia of Genes and Genomes pathways in differentially expressed genes.

To begin to clarify how morphine may inhibit HCC cell growth, we compared the transcriptome of QGY-7703 cells before and after treatment with morphine using a GeneChip array. We identified 766 genes up-regulated and 311 genes down-regulated by morphine. Pathway enrichment analysis suggested that genes differentially expressed after morphine treatment were involved mainly in aminoacyl-tRNA biosynthesis; glycine, serine and threonine metabolism; natural killer cell-mediated cytotoxicity; and MAPK signaling (Figure 2).

### DDX49 is down-regulated by morphine treatment of HCC cells *in vitro*

**Figure 3:**
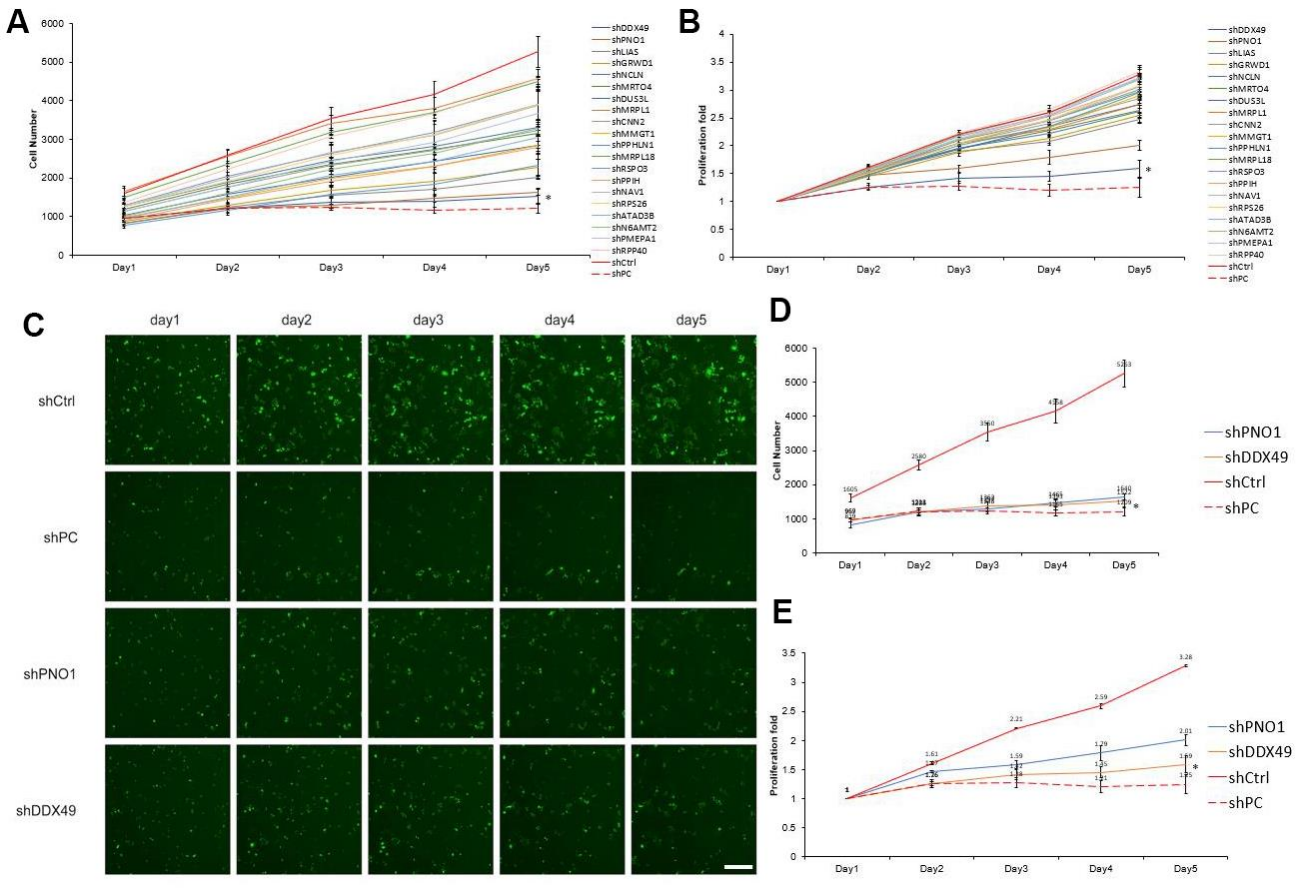
**Screening of 20 differentially expressed genes for anti-HCC effects *in vitro*.** (**A**, **B**) The 20 genes most down-regulated by morphine treatment were knocked down using lentivirus-delivered shRNA, and effects on QGY-7703 cell proliferation were examined. A) Shows the cells number after the 20 genes were knock down. B) Shows the cells proliferation fold changed after the 20 genes were knock down. (**C**–**E**) Cells were treated with a plasmid encoding a negative-control shRNA (shCtrl) or plasmids encoding shRNAs targeting PNO1 (shPNO1) or DDX49 (shDDX49). shPC as the protooncogene X specific-targeting shRNA. Then the cells were analyzed for five days by fluorescence microscopy, and the data were used to measure proliferation. Scale bar, 50 μm. *P < 0.01 compare to the control group.

To identify genes through which morphine may exert its anti-HCC effects, we analyzed further the 20 most down-regulated genes, based on fold change (Supplementary Table 1). Celigo assay results showed that knocking down the DDX49 gene suppressed QGY-7703 proliferation by 2.07-fold relative to the negative control, while knocking down PNO1 suppressed proliferation by 1.63-fold (Figure 3). We focused on DDX49 in subsequent experiments because of its stronger effect.

### Knocking down DDX49 inhibits HCC tumor growth *in vivo*

**Figure 4:**
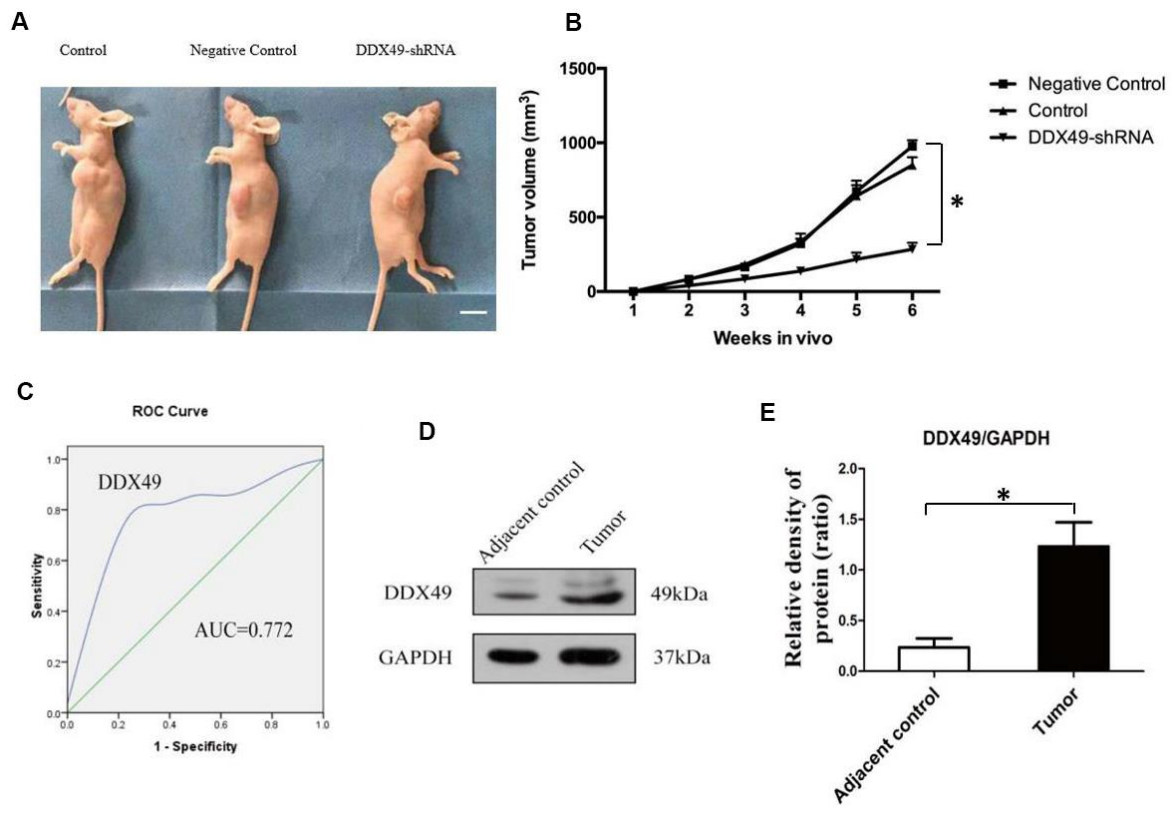
**DDX49 expression and its effects on HCC xenografts in mice and HCC in patients.** (**A**, **B**) QGY-7703 cells were transfected with DDX49-shRNA or negative-control shRNA, then injected into nude mice (N=8 animals/group). Control mice were nude mice inject with tumor cells. Scale bar, 1 cm. After 6 weeks, tumor volume was measured. (**C**) DDX49 expression in HCC patients was used to differentiate tumor tissue and normal liver tissue. (**D**, **E**) Western blot analysis of DDX49 in paired samples of tumor and normal liver tissue from HCC patients. *P < 0.01 compared to the control group.

QGY-7703 cells were transfected with DDX49-shRNA or negative-control shRNA (control) and injected into nude mice. Analysis of tumor size after 6 weeks showed that knocking down DDX49 led to significantly smaller tumors (Figure 4A, *P* < 0.05).

### DDX49 may regulate MAPK activity in HCC

**Figure 5:**
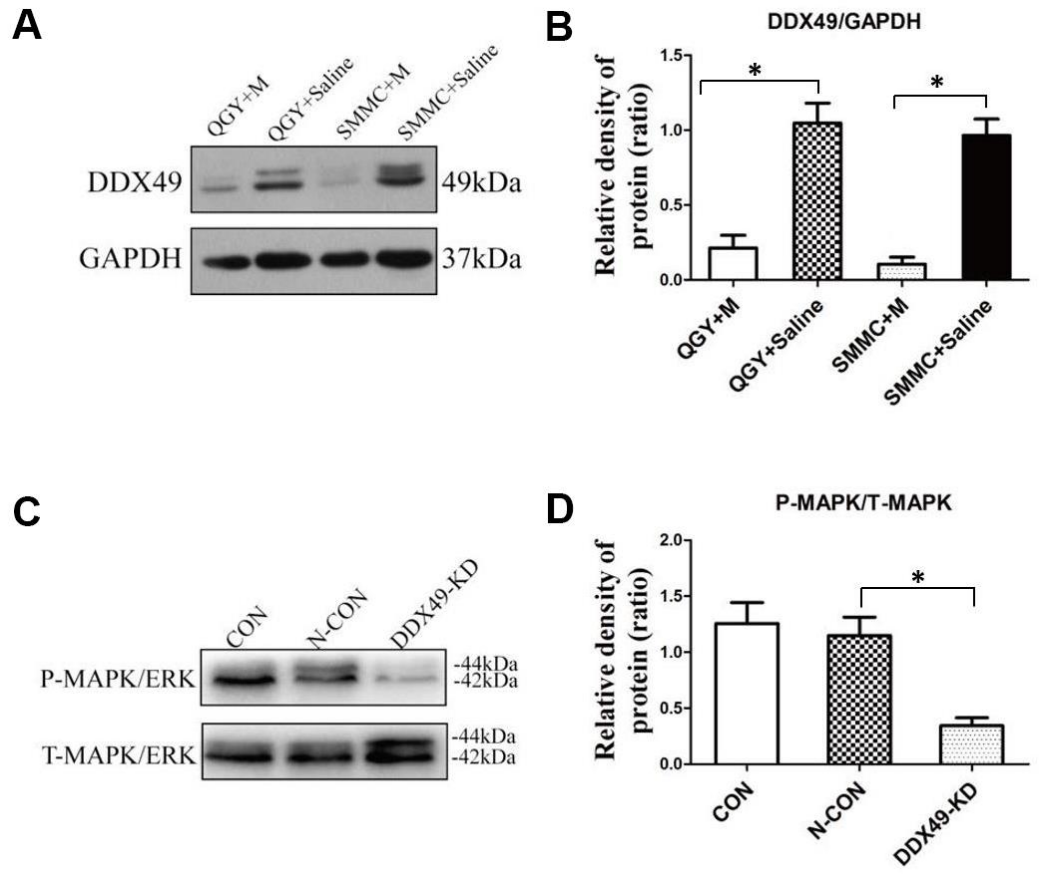
**DDX49 expression after morphine treatment and its impact on MAPK signaling.** QGY-7703 and SMMC-7721 cells were treated with morphine (10μM) or saline for 48 h. (**A**, **B**) Effect of morphine (10μM) on levels of DDX49 in QGY-7703 and SMMC-7721 cells. (**C**, **D**) Effect of DDX49 expression on levels of phosphorylated MAPK (P-MAPK) and levels of total MAPK (T-MAPK). *P < 0.01 compared to the respective control group. CON cells were derived cells without any treating. N- CON cells were knock down using negative control vector. DDX49-KD cells were knock down of DDX49 using lentivirus.

MAPK signaling pathways often play a role in oncogenesis [9], and we found that genes differentially expressed in QGY-7703 cells after morphine treatment were enriched in the KEGG pathway of MAPK signaling. Both of MAPK and DDX49 are located in nucleus, so whether DDX49 have an impact on MAPK raised our interesting. Indeed, we found that knocking down DDX49 led to lower levels of phosphorylated MAPK (Figure 5C), which correlates with less MAPK-mediated signaling [9].

### DDX49 is up-regulated in HCC tumors from patients

Comparison of paired tumor samples and normal liver tissue from patients with HCC in Barcelona Clinic Liver Cancer (BCLC) stage A showed that DDX49 levels were significantly higher in cancerous tissues (Figure 4D, 4E). Next we examined whether DDX49 might be useful as an HCC biomarker. The area under the receiver operating characteristic curve for differentiating tumor from normal liver tissue was 0.772 (P=0.037), giving sensitivity of 71.4% and specificity of 87.5% (Figure 4C). The Youden index was 0.589.

### Morphine down-regulates DDX49 in HCC cells *in vitro*

Morphine (10μM) significantly down-regulated DDX49 in HCC cells (Figure 5A, P < 0.05).

### Overexpressing DDX49 reverses the anti-HCC effects of morphine *in vitro*, while knocking down DDX49 reinforces them

**Figure 6:**
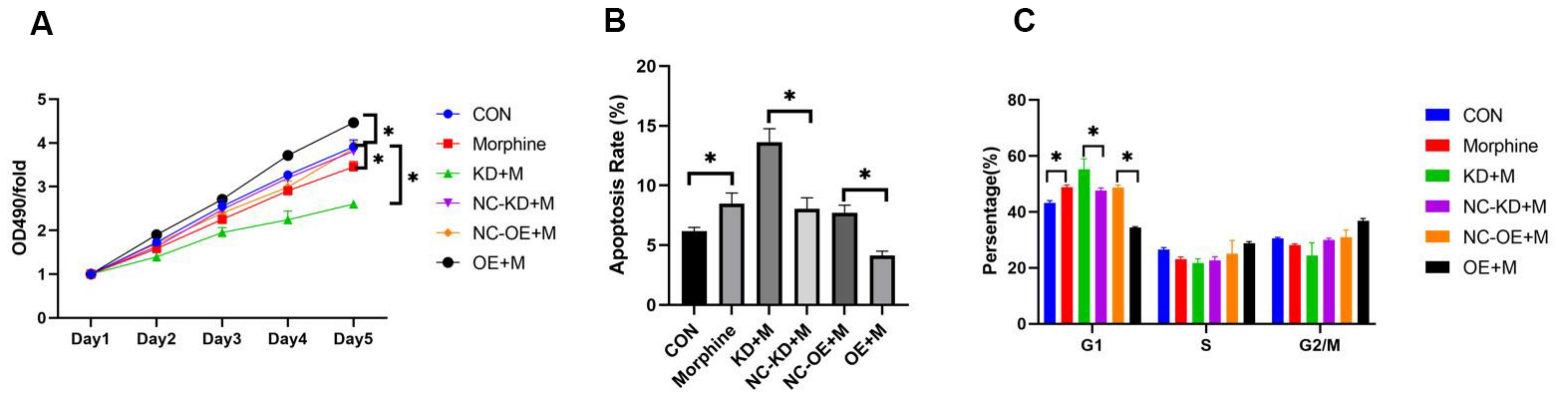
**Effect of DDX49 on HCC cell lines treated with morphine.** QGY-7703 cells were transfected with LV-DDX49-KD (KD) or LV-DDX49-OE (OE) or the corresponding negative control lentivirus (NC-KD, NC-OE), then treated with morphine (10μM) for 5 days. (**A**) Proliferation was assessed using the MTT assay. OD490, optical density at 490 nm. (**B**) Apoptosis rates in the different groups were assessed using flow cytometry. (**C**) Cell cycle in the different groups were assessed using flow cytometry. *P < 0.01 compared to the respective control group. CON cells were derived cells without any treating. Morphine QGY-7703 cells were treated with morphine (10μM) for 48h. NC-KD+M cells were knock down using negative control vector and treat with morphine for 48h. KD+M cells were knock down of DDX49 using lentivirus and treat with morphine for 48h; OE+M cells were transfected with over-expression of DDX49 lentivirus vector and treat with morphine with 48h; NC-OE+M cells were transfected over-expression of DDX49 negative control vector and treat with morphine for 48h.

To identify the functional role of DDX49 in HCC tumorigenesis, we infected QGY-7703 cells with either DDX49 overexpression vector (LV-DDX49-OE) or lentivirus mediated DDX49 silencing vector (LV- DDX49-KD) to increase or knock down the DDX49 expression, respectively. Then cells were treated with morphine for 48h. Overexpression of DDX49 in HCC cells reversed cell proliferation inhibition effects of morphine compare to negative control vector-transfected (Figure 6A). HCC cells with DDX49 knocking down showed significantly proliferation inhibition which was accessed by MTT assay. At day 5 DDX49 deletion inhibit cell growth up to 68.7% compare to the negative control (Figure 6A, *P* < 0.05).

To further study the role of morphine in the regulation of cell apoptosis, cells were treated with LV-DDX49-OE or LV- DDX49-KD. Cells with DDX49 over-expressing lentivirus decreased cell apoptosis compare to the negative control virus, P < 0.01. While cells with DDX49 knocking down increased cell apoptosis (Figure 6B). Compare with negative control vector-transfected group, knocking down of DDX49 increase the cells cycle arrest in G1. Whereas overexpressing DDX49 lentivirus decreased the cells cycle arrest in G1 (Figure 6C). There were no significant different between knocking down of DDX49 and negative control in the cells cycle of S, G2 and M. (Figure 6C).

### Knocking down DDX49 inhibits colony formation by HCC cells *in vitro*

**Figure 7:**
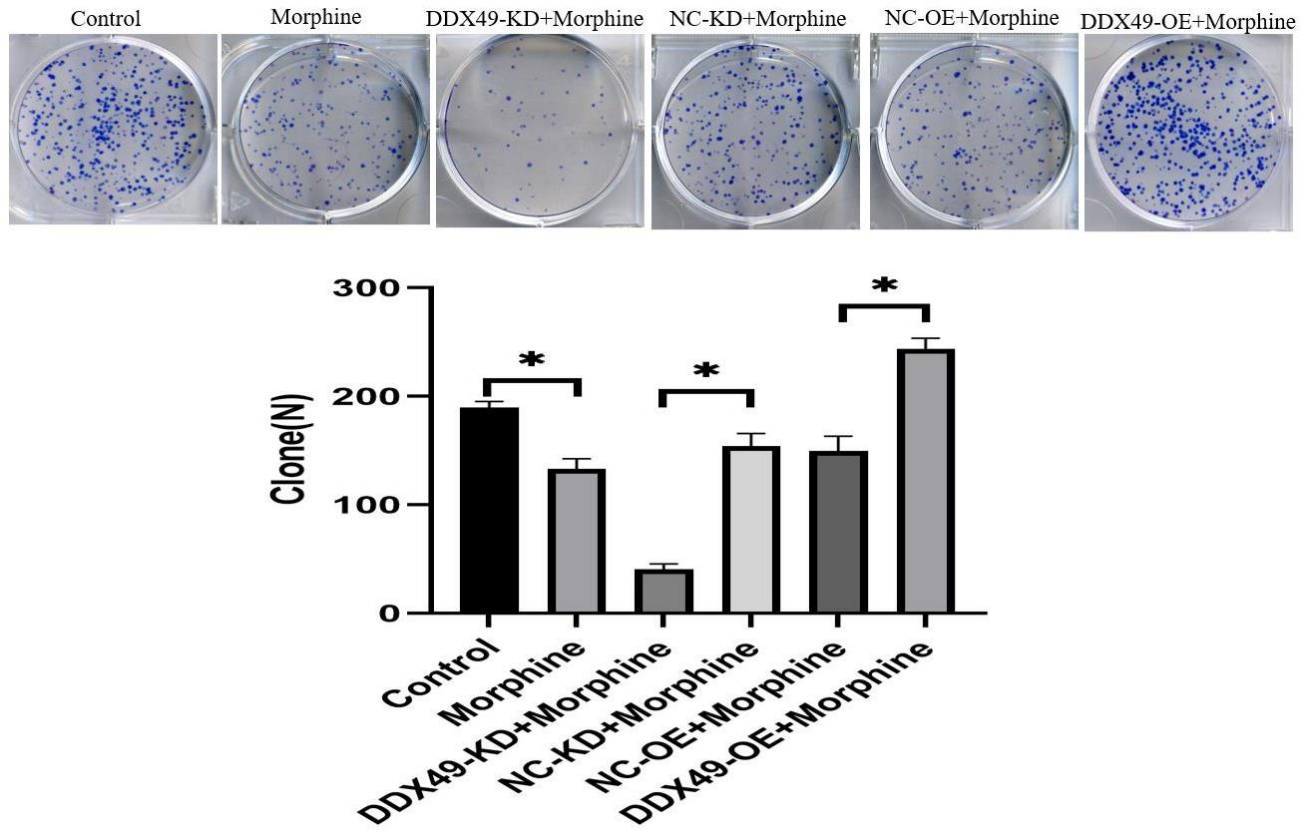
**Effect of DDX49 on cell clone formation after HCC cells treat with morphine.** Cell colony formation after DDX49 depletion/overexpression treat with morphine(10μM). Morphine QGY-7703 cells were treated with morphine (10μM) for 48h. *P < 0.01 compared to the respective control group.

Knocking down DDX49 in QGY-7703 cells significantly reduced their ability to form colonies, while overexpressing DDX49 remarkably increased their ability to form colonies (Figure 7).

### Knocking down DDX49 inhibits HCC metastasis *in vitro*

**Figure 8:**
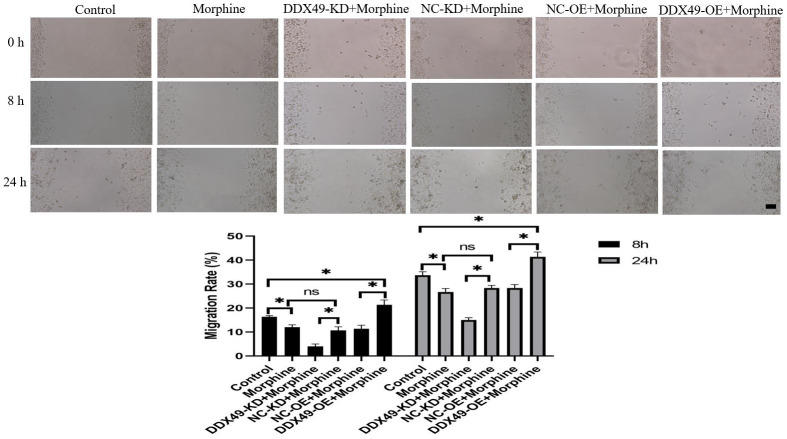
**Effect of DDX49 on migration after HCC cells treat with morphine.** Phase micrographs of migration HCC cells with DDX49 knock down or over expression in wound healing assay after exposure to morphine(10μM). Scale approximately 500 μm. Morphine QGY-7703 cells were treated with morphine (10μM) for 48h. *P < 0.05 compare to the respective control group. NS non-significant different.

**Figure 9:**
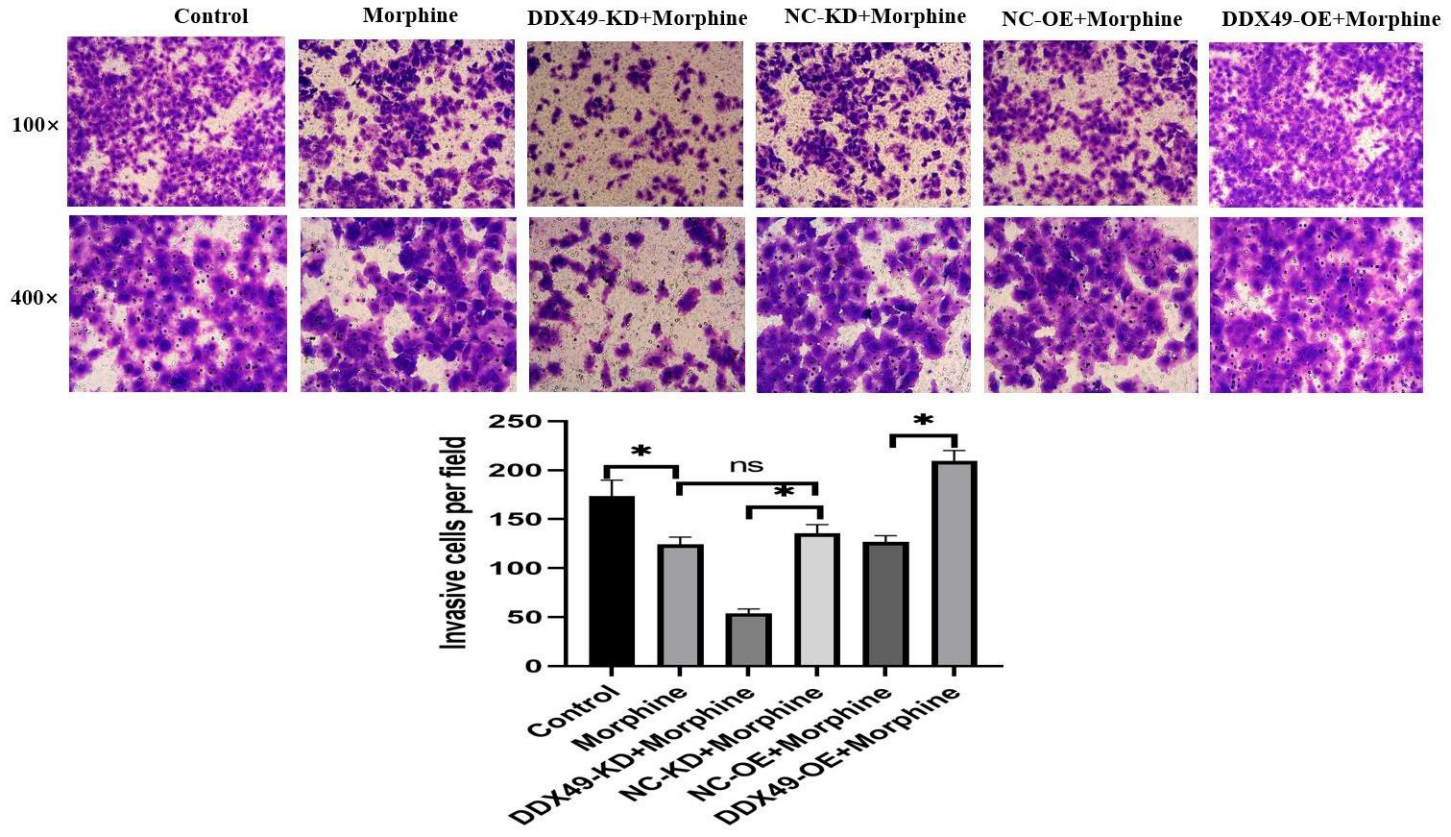
**Effect of DDX49 on invasion after HCC cells treat with morphine.** Phase micrographs of invading HCC cells with DDX49 knock down or over expression in Matrigel invasion assay after exposure to control or morphine. Morphine QGY-7703 cells were treated with morphine (10μM) for 48h. *P < 0.05 compare to the respective control group. NS non-significant different.

Our data showed that DDX49 has a direct regulation on cell proliferation. This prompted us to investigate whether DDX49 has a regulation role in cancer metastasis. We subsequently investigated the migration and invasion of QGY-7703 cells following the DDX49 deletion or overexpression and then treat with morphine for 48h. We found that DDX49 deletion decreased cells migration (Figure 8) and invasion (Figure 9). Overexpressing DDX49 reversed the cells migration (Figure 8) inhibition effect of morphine, and analogical results can be observed in invasion assay (Figure 9). These data suggested that DDX49 may increase the capacity of migration and invasion in HCC cells.

## DISCUSSION

Improving pain control in cancer patients is a priority for patients and clinicians. Morphine is a first-line opioid analgesic for patients with various types of cancer, including HCC [10]. Several studies suggest that, in addition to relieving pain, morphine can inhibit cancer cell growth and proliferation, based in studies *in vitro* and in xenograft-bearing animals [11–13]. The present study extends this literature by demonstrating these anti-cancer effects against HCC, confirming previous studies that morphine can significantly inhibit proliferation of human HCC QGY-7703 and SMMC-7721 cells.

Moreover, morphine has been shown to induce apoptosis and cell cycle arrest [14–17] in various types of cancer [18]. Consistently, we found that morphine promoted apoptosis and induced G0/G1 cell cycle arrest in a dose-dependent manner in two HCC cell lines. How morphine exerts these effects is unclear. Previous work has suggested that the effects may involve heregulin-stimulated ErbB signaling in the case of breast cancer cells [11] or opioid receptors in the case of neuroblastoma cells [19]. Here we used GeneChip analysis to screen for genes that may help mediate the anti-cancer effects of morphine in HCC. The genes whose expression was altered by morphine treatment appear to participate primarily in lysosomes, endocytosis, antigen processing, natural killer cell-mediated cytotoxicity and MAPK signaling. We focused on the DEAD box protein DDX49 as one of the genes whose expression was most altered by morphine. Other members of the DEAD box family have already been implicated in cancer [20]. For example, overexpression of DDX5 (also called p68) can transform healthy cells, and it is up-regulated in colorectal cancer [21–24]. Another example is DDX3, which normally inhibits cell growth by activating CDKN1A (also called p21WAF1 or CIP1) but is down-regulated in hepatic cancers [25] and whose translocation from the nucleus to the cytoplasm has been implicated in cutaneous squamous cell carcinoma [26, 27]. Our results suggest that, conversely, DDX49 is normally expressed at low levels in healthy liver but is up-regulated in HCC. Knocking down DDX49 inhibited HCC tumor growth and metastasis, while overexpressing it reinforces tumor growth and metastasis. Analogously, another group reported that up-regulation of DDX49 may be linked to increase cell proliferation in HeLa DDX49 overexpression cancer cells [28].

We found that morphine antagonized the effects of DDX49 overexpression while reinforced the effects of DDX49 knockdown. These effects of morphine likely reflect the drug’s ability to down-regulate DDX49 expression. Our results suggest that morphine inhibits HCC progression in part by down-regulating DDX49.

Our experiments suggest that one way in which morphine inhibits HCC is to reduce levels of activated (phosphorylated) MAPK, which presumably reduces downstream activation of target proteins that remain to be identified. MAPKs direct cellular responses to a diverse array of stimuli, such as mitogens, osmotic stress, heat shock and proinflammatory cytokines. Further studies should explore in detail what downstream processes are affected by morphine in HCC. They should also build on our finding that DDX49 may increase MAPK expression in HCC.

Whatever the mechanism(s) through which morphine and DDX49 affect HCC, we found that DDX49 expression level differentiated HCC tissue from adjacent healthy liver tissue with good sensitivity and specificity, based on receiver operating characteristic curves. Further study should explore the diagnostic potential of this biomarker, alone and in combination with other biomarkers such as AFP.

Our results should be interpreted with caution given several limitations in the study. The morphine concentrations that we used differ from physiological concentrations in humans. In fact, animal studies have given conflicting results about the effects of morphine on cancer: for example, one study showed that 40 μM morphine promoted glioblastoma proliferation [29], while morphine concentrations of 0.35–350 μM did not affect melanoma cell proliferation and a concentration of 3500 μM inhibited proliferation [12]. The literature suggests that morphine’s effects on cancer may depend on its concentration and the type of cancer, which highlights the need for more careful animal modelling and, ultimately, studies in humans.

Despite these limitations, our study provides evidence that DDX49 can help drive HCC and that morphine may inhibit disease progression in part by down-regulating DDX49.

### Statement of written informed consent

All authors of the study agreed to submit the manuscript to this journal.

## Supplementary Material

Supplementary Figure 1

Supplementary Table 1
