# Supplementary material for: Morphine may act via DDX49 to inhibit hepatocellular carcinoma cell growth
Source: Aging (Albany NY). 2021 May 5;13(9):12766–79. doi: 10.18632/aging.202946 (PMC8148497; doi:10.18632/aging.202946)
Supplement: Supplementary Figure 1 [file aging-13-202946-s001.pdf]

SUPPLEMENTARY FIGURE

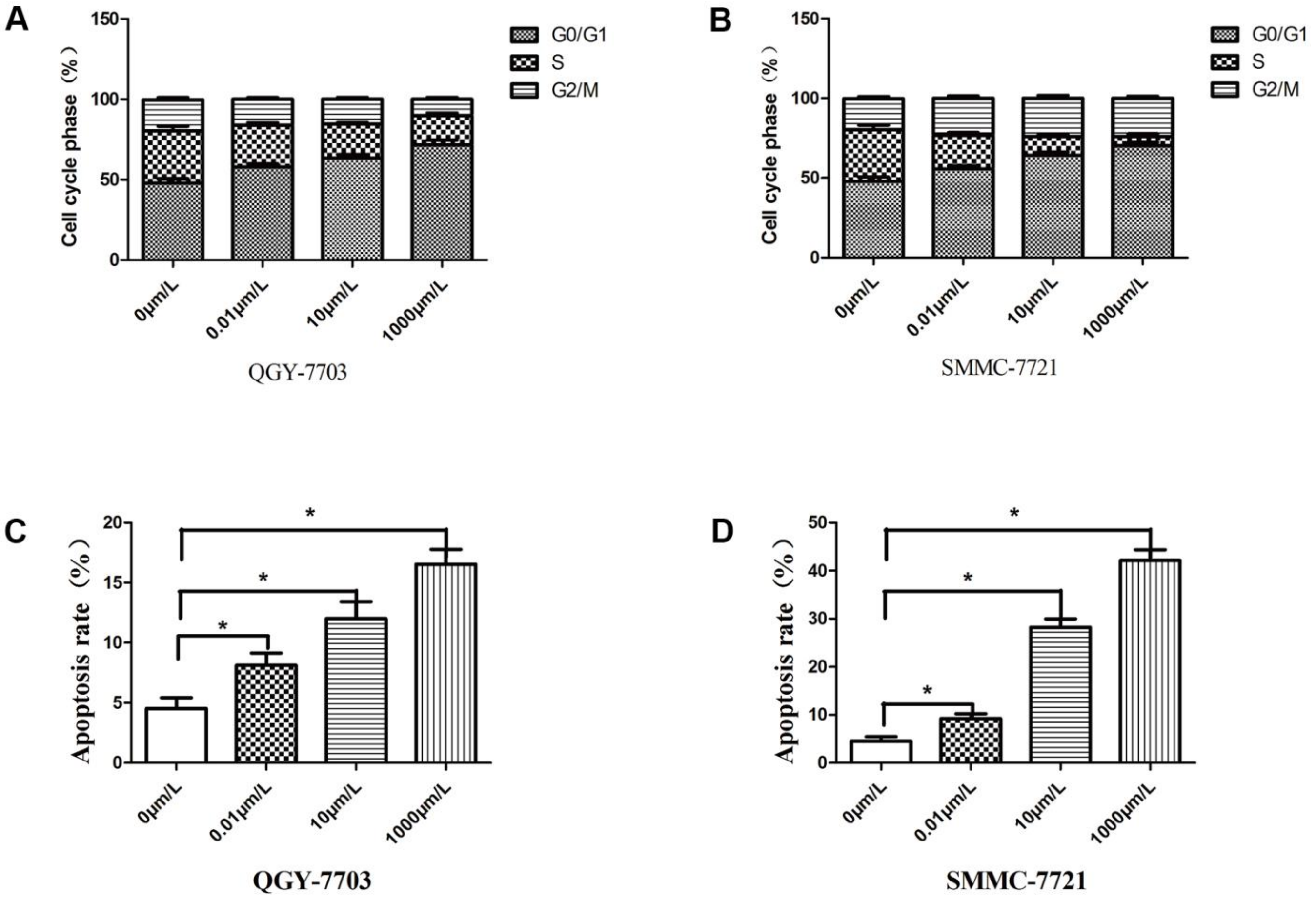

Supplementary Figure 1. Effect of morphine on HCC cell cycle and apoptosis *in vitro*. The two HCC cell lines QGY-7703 and SMMC-7721 were treated with morphine at the indicated dose for 48 h, then analyzed for cell cycle distribution and apoptosis by flow cytometry. (**A**, **B**) Cell cycle distribution of. (**C**, **D**) Apoptosis rates in the cell lines of QGY-7703 and SMMC-7721 were assessed using flow cytometry. * P< 0.05 compared to the untreated control.
