# Supplementary material for: Morphine may act via DDX49 to inhibit hepatocellular carcinoma cell growth
Source: Aging (Albany NY). 2021 May 5;13(9):12766–79. doi: 10.18632/aging.202946 (PMC8148497; doi:10.18632/aging.202946)
Supplement: Supplementary Table 1 [file aging-13-202946-s002.pdf]

SUPPLEMENTARY TABLE

Supplementary Table 1. The top 20 down-regulated genes list after morphine treatment.

Gene name	Fold change	Regulation	P-value
DDX49	6.07	Down	0.000180206
PNO1	3.63	Down	0.001249399
LIAS	1.80	Down	0.000345558
GRWD1	1.56	Down	0.003242646
NCLN	1.66	Down	4.75146E-05
MRTO4	1.52	Down	5.06618E-05
DU53L	1.70	Down	0.002851743
MRPL1	1.67	Down	0.000543709
CNN2	1.51	Down	0.00049281
MMGT1	1.61	Down	0.003791277
PPHLN1	1.50	Down	0.000185112
MRPL18	1.57	Down	0.008402954
RSPO3	1.74	Down	0.001760566
PPIH	1.60	Down	0.00057681
NAV1	1.53	Down	0.00107331
RPS26	1.51	Down	0.008459099
ATAD3B	1.51	Down	0.000197995
N6AMT2	1.53	Down	0.001307202
MEPA1	1.62	Down	0.000100151
RPP40	1.55	Down	3.43857E-05
